# Change in brain amyloid load and cognition in patients with amnestic mild cognitive impairment: a 3-year follow-up study

**DOI:** 10.1186/s13550-022-00928-5

**Published:** 2022-09-05

**Authors:** Elina Rauhala, Jarkko Johansson, Mira Karrasch, Olli Eskola, Tuula Tolvanen, Riitta Parkkola, Kirsi A. Virtanen, Juha O. Rinne

**Affiliations:** 1 Clinical Neurosciences, Faculty of Medicine, Turku University Hospital, University of Turku and Neurocenter, Turku, Finland; 2 Turku PET Centre, Turku University Hospital, Turku, Finland; 3 Department of Radiation Sciences, Umeå University, Umeå, Sweden; 4 Department of Psychology, Åbo Akademi University, Turku, Finland; 5 Turku PET Centre, University of Turku, Turku, Finland; 6 Department of Medical Physics, Turku University Hospital, Turku, Finland; 7 Department of Radiology, University of Turku and Turku University Hospital, Turku, Finland; 8 InFLAMES Research Flagship Center, University of Turku, Turku, Finland

**Keywords:** Flutemetamol, Positron emission tomography, Mild cognitive impairment, Alzheimer, Amyloid PET, Follow-up, Cognition

## Abstract

**Background:**

Our aim was to investigate the discriminative value of ^18^F-Flutemetamol PET in longitudinal assessment of amyloid beta accumulation in amnestic mild cognitive impairment (aMCI) patients, in relation to longitudinal cognitive changes.

**Methods:**

We investigated the change in ^18^F-Flutemetamol uptake and cognitive impairment in aMCI patients over time up to 3 years which enabled us to investigate possible association between changes in brain amyloid load and cognition over time. Thirty-four patients with aMCI (mean age 73.4 years, SD 6.6) were examined with ^18^F-Flutemetamol PET scan, brain MRI and cognitive tests at baseline and after 3-year follow-up or earlier if the patient had converted to Alzheimer´s disease (AD). ^18^F-Flutemetamol data were analyzed both with automated region-of-interest analysis and voxel-based statistical parametric mapping.

**Results:**

^18^F-flutemetamol uptake increased during the follow-up, and the increase was significantly higher in patients who were amyloid positive at baseline as compared to the amyloid-negative ones. At follow-up, there was a significant association between ^18^F-Flutemetamol uptake and MMSE, logical memory I (immediate recall), logical memory II (delayed recall) and verbal fluency. An association was seen between the increase in ^18^F-Flutemetamol uptake and decline in MMSE and logical memory I scores.

**Conclusions:**

In the early phase of aMCI, presence of amyloid pathology at baseline strongly predicted amyloid accumulation during follow-up, which was further paralleled by cognitive declines. Inversely, some of our patients remained amyloid negative also at the end of the study without significant change in ^18^F-Flutemetamol uptake or cognition. Future studies with longer follow-up are needed to distinguish whether the underlying pathophysiology of aMCI in such patients is other than AD.

## Introduction

It is known that about 60% of subjects with amnestic Mild Cognitive Impairment (aMCI) will convert to Alzheimer's disease (AD) [1]. Elevated brain amyloid load has been associated with subtle, but slightly more marked cognitive decline than what would be expected solely in “normal aging” [2]. Follow-up studies have shown differences in cognitive performance [3, 4] and brain amyloid accumulation [5–7] between those who convert to AD and those who do not. Those patients who converted to AD had lower cognitive measurements and higher uptake of amyloid binding ^11^C-PIB-PET ligand referring to greater accumulation of amyloid at baseline.

^18^F-Flutemetamol is a PET ligand which has high affinity for amyloid *β*. The use ^18^F-Flutemetamol ligand permits in vivo detection of amyloid deposition in the brain. Brain ^18^F-Flutemetamol uptake has been shown to be associated with the amount of beta-amyloid plaques [8–10]. In addition, it has been shown to differentiate between patients with AD and healthy controls [11]. In aMCI patients, ^18^F-Flutemetamol uptake has been positive approximately in half of the cases [11–13]. Amyloid positivity in ^18^F-Flutemetamol PET, low hippocampal volume, and cognitive status corresponded with a high probability of risk of progression from aMCI to probable AD within 36 months [13]. In earlier studies, the follow-up has performed with cognitive tests without amyloid PET scanning.

The aim of this study was to examine whether there is increase in ^18^F-Flutemetamol uptake during the course of aMCI and whether this increase is different between those aMCI subjects who were amyloid positive vs amyloid negative at baseline. Our study design also enabled us to analyze association between changes in brain amyloid load and cognition over time.

## Material and methods

### Subjects

Demographics of the patients are shown in Table 1. Altogether 34 patients were included in the study (23 men and 11 women). The mean age of the patients was 73.4 ± 6.6 (mean ± SD) at baseline. All patients met the criteria of amnestic MCI [13], and all patients gave their written informed consent, which was obtained according to the requirements of the Declaration of Helsinki. The study protocol was approved by the Ethics Committee of Hospital District of Southwest Finland.

**Table 1:** Demographics of the study participants

	All	Amyloid positive	Amyloid negative
Patients at baseline	*N* = 34	*N* = 15	*N* = 19
Mean age at baseline	73.4 ± 6.6	75.9 ± 3.7	71.4 ± 7.7
Age range at baseline	60–86	68–82	60–86
Mean education (years)	12.7 ± 2.6	12.5 ± 2.8	12.8 ± 2.5
Range education	9–16	9–16	9–16
Males at baseline (%)	*N* = 23 (67.6)	*N*= 10 (66.7)	*N* = 13 (68.4)
Females at baseline (%)	*N*= 11 (32.4)	*N*= 5 (33.3)	*N* = 6 (31.6)

Age and education are presented as mean ± standard deviation (SD). *N* number of individuals

The patients were assessed with ^18^F-Flutemetamol PET scan, brain MRI and cognitive tests. Cognitive tests were administered yearly until conversion to Probable Alzheimer’s Disease or the end of the 3-year follow-up. Probable Alzheimer’s disease was diagnosed when the patients fulfilled the National Institute of Neurological and Communicative Disorders and Stroke-Alzheimer’s Disease and Related Disorders Association criteria as well as DSM-IV criteria for dementia of the Alzheimer type. MRI and ^18^F-flutemetamol PET were repeated after the subject had converted to AD or after 3 years had elapsed from the first ^18^F-Flutemetamol PET scan. ^18^F-Flutemetamol composite cortical uptake value ratio > 1.4 was used as a cut-off value of amyloid positivity [14]. Patients with amyloid-positive and amyloid-negative ^18^F-Flutemetamol scans at baseline did not differ regarding education and age at baseline (Table 1). Because the study was ongoing for several years, the PET scanner in the PET Centre changed during the study. Therefore, 14 patients were examined with one and 20 with the other scanner (see below for details). Baseline and follow-up scan was done with the same scanner in all but in 2 patients.

From the original 34 participants, seven dropped out the study after first scan and cognitive tests. Four of those 7 patients had positive baseline ^18^F-Flutemetamol PET scan. Five patients dropped out due to worsening of clinical condition, one participant died because of acute pulmonary embolism and one withdrew the consent due to personal reasons. Altogether 4 participants converted to AD during the study, 3 of them were amyloid positive and one was negative both at baseline and follow-up.

Additional 2 patients were excluded from the PET-analyses due to technical problems in image acquisition. Thus, both baseline and follow-up ^18^F-Flutemetamol PET data were available from 25 participants.

### PET and MRI imaging

All patients underwent a ^18^F-Flutemetamol PET scan. They received approximately 185 MBq of intravenous ^18^F-Flutemetamol (187.8 ± 18.7 MBq, mean ± SD) and 90 min later underwent a 30-min brain scan. PET scan was done at baseline and approximately after 3-year follow-up or earlier if the patient converted to AD. The mean ^18^F-Flutemetamol scanning interval was 2.9 ± 0.4 years. Two patients’ follow-up PET scans were excluded from the analysis due to inadequate cerebellar imaging precluding reliable determination of reference region; one had positive and the other negative amyloid status at baseline.

First, the ECAT EXACT HR + (CTI/Siemens, Knoxville, TN, US) was used for PET imaging in 3D mode. The scanner has an axial field of view of 15.5 cm and a patient port of 56.2 cm, and physical performance evaluations of the scanner have shown radial and tangential average spatial resolution of 4.39 mm FWHM and axial resolution of 5.10 mm FWHM (16).

Later, both PET scans and follow-up scan in two patients, which were examined with ECAT scanner at baseline, were performed with GE Discovery 690 (GE Healthcare, Waukesha, WI, US) PET scanner because at that time ECAT EXACT HR + PET scanner was not in use anymore. GE Discovery 690 is a hybrid PET/CT scanner with the axial field of view of 15.7 cm and the patient port is 70.0 cm. Physical performance evaluations of the scanner have shown radial and tangential average spatial resolution of 4.70 mm FWHM and axial resolution of 4.74 mm FWHM [15].

All patients underwent brain MR imaging at baseline and after 3-year follow-up or earlier if the patient was converted to AD. MR imaging was done on Philips 1.5 T Gyroscan Intera CV Nova Dual MR scanner (Philips Medical Systems, Best, The Netherlands). A head coil (Philips Medical Systems, Best, The Netherlands) was used in the measurement. Whole-brain T1-weighted three-dimensional fast field echo data with 1-mm isotropic voxels were acquired in the transverse plane (acquisition parameters: repetition time 25 ms, echo time 5.5 ms, flip angle 30°, field of view 256 × 256 mm) yielding at least 160 contiguous slices to cover the whole head. In addition, routine axial T2-weighted and coronal FLAIR images were obtained.

### ^***18***^***F-Flutemetamol image analysis***

To obtain quantitative regional values of ^18^F-Flutemetamol uptake, an automated volume of interest (VOI) analysis was conducted as described previously [16]. Briefly, parametric images representing ^18^F-Flutemetamol uptake in each pixel were calculated as a region-to-cerebellum ratio of the radioactivity concentration over 90–120 min after ^18^F-Flutemetamol injection. In order to compensate for head motion during PET imaging, the three ten-minute frames of ^18^F-Flutemetamol uptake were registered to each other prior to parametric image calculation. A rigid image registration algorithm implemented in statistical parametric mapping (SPM, version 8) software in MATLAB was employed. The motion-corrected data were subsequently summed and co-registered with subject-specific T1-weighted MR images in native space. Finally, the unified segmentation algorithm in SPM8 [17] was used for spatially normalizing the MR and PET images into the standard Montreal Neurological Institute (MNI) coordinate space. Automated VOI-delineation in the MNI space was defined on the basis of automated anatomical labeling [18] atlas, and gray matter masking using individual gray matter segments thresholded at 25%. Standard VOIs of cerebellar gray matter, frontal cortex, parietal cortex, lateral temporal cortex, anterior and posterior cingulate and precuneus were used in the analysis. A composite cortical amyloid uptake score was formed by combining the VOIs of frontal, parietal and lateral temporal cortices and posterior cingulate, similar to our earlier investigations [19]. Average regional VOI-values were extracted from spatially normalized ^18^F-Flutemetamol uptake ratio images within the above-mentioned VOIs, except in the cerebellar cortex which served as a reference region.

### Cognitive testing

General cognition was assessed using Mini-Mental State Examination (MMSE). A subset of tests defined in Wechsler Memory Scale revised (WMS-R [20]) were conducted to assess immediate recall (logical memory I), delayed recall (logical memory II) and verbal fluency. All tests were delivered yearly to follow-up conversion to AD. Scores from the test occasions closest in time with baseline and follow-up PET imaging were used in the analysis.

### Statistical analysis

VOI-based statistical analysis was conducted using *R* (version 4.0.3). Descriptive statistics included mean and standard deviations of continuous variables and counts for categorical variables. Student’s t test was employed for paired and Welcher two-sample t test for unpaired analysis. Pearson product moment correlation coefficients were used for examining the relationships between cognitive and imaging measures. Significance level was set at *p* < 0.05 (trend-level *p* < 0.1).

Confirmatory voxel-wise analysis was conducted using SPM8. Briefly, the spatially normalized ^18^F-Flutemetamol uptake ratio images were first smoothed using a kernel of 8 mm (FWHM; 3D) and, secondly, analyzed with paired *t* test to detect group-level changes in ^18^F-Flutemetamol uptake ratios over time. The resulting voxel-wise maps of *T*-statistics were inspected for regional changes of ^18^F-Flutemetamol uptake ratios using a liberal threshold of *T* ≥ 2.5, corresponding to approximately *p* < 0.01 (uncorrected). The use of a liberal threshold allowed detection of clusters that were potentially uncovered using VOI-analysis.

## Results

**Fig. 1:**
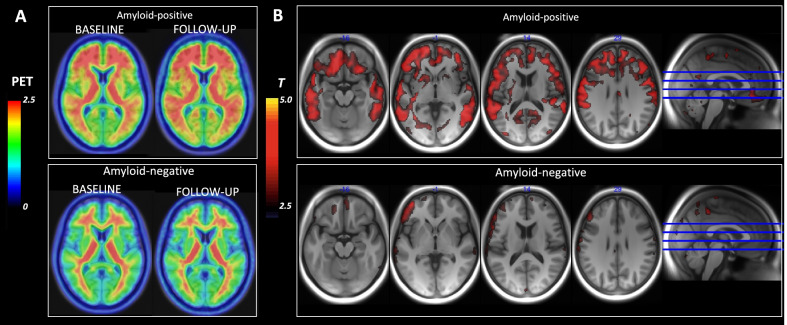
**A** Average voxel-wise maps of baseline and follow-up.^18^F-Flutemetamol PET uptake ratios (warmer colors represent higher uptake ratios) in patients with amyloid-positive (*N* = 15/10) and amyloid-negative (*N* = 19/15) baseline scans. Patients with amyloid-positive baseline scans showed increased cortical 18F-Flutemetamol uptake. In contrast, in patients with amyloid-negative baseline scans, negligible 18F-Flutemetamol uptake was seen in gray matter at baseline or follow-up. **B** Voxel-wise statistical mapping was conducted to assess longitudinal change. Widespread clusters of positive change were observed in amyloid-positive (top panel), but not in amyloid-negative patients (bottom panel). Statistical parametric maps are expressed in *T*-values using a threshold *T* ≥ 2.5 (cluster-forming threshold zero), corresponding to approximately *p* < 0.01 (uncorrected)

The patients were classified as either positive (uptake ratio ≥ 1.4) or negative (uptake ratio < 1.4) based on the ^18^F-Flutemetamol composite cortical uptake score (see Table 2). At baseline, 15 patients (44%) had a positive and 19 patients (56%) had a negative ^18^F-Flutemetamol scan. The mean ^18^F-Flutemetamol composite score at baseline was 1.49 ± 0.36 and at follow-up 1.54 ± 0.39. Statistically significant increases in ^18^F-Flutemetamol uptake ratio were observed in the composite score, as well as in the prefrontal cortex, parietal cortex, lateral temporal cortex, and precuneus, for all patients together, as well as for patients with positive or negative baseline scans alone (Table 2). However, the increase in ^18^F-Flutemetamol uptake during follow-up was larger in patients who were ^18^F-Flutemetamol positive at baseline than in those who were negative [increase 0.15 ± 0.10 *p* < 0.001 and 0.05 ± 0.08 *p* < 0.08, respectively, Welcher two-sample *t* test *t*(16) = 2.64*]. Voxel-wise maps of ^18^F-Flutemetamol uptake ratios at baseline revealed a pattern of more wide-spread cortical uptake in amyloid-positive patients as compared to the amyloid-negative patients (Fig. 1a). Furthermore, voxel-wise statistical parametric mapping showed large clusters of increased follow-up ^18^F-Flutemetamol uptake ratios in patients with amyloid-positive baseline scans in the prefrontal, parietal, and lateral temporal regions, in agreement with the VOI-analysis (Fig. 1b). No significant clusters of increased ^18^F-Flutemetamol uptake ratios were observed in amyloid-negative patients (Fig. 1c).

**Table 2:** PET imaging results stratified by test time and amyloid status

	All	Amyloid positive	Amyloid negative	Group difference
Composite score baseline	1.49 ± 0.36 (*N* = 34)	1.84 ± 0.24 (*N* = 15)	1.21 ± 0.06 (*N* = 19)	*t*(15) = 10.00***
Composite score follow-up	1.54 ± 0.39 (*N* = 25)	1.96 ± 0.24 (*N* = 10)	1.26 ± 0.12 (*N* = 15)	*t*(12) = 8.65***
Composite score delta	0.09 ± 0.10***	0.15 ± 0.10**	0.05 ± 0.08*	*t*(16) = 2.64*
Prefrontal cortex baseline	1.52 ± 0.39 (*N* = 34)	1.91 ± 0.25 (*N* = 15)	1.22 ± 0.06 (*N* = 19)	*t*(15) = 10.29***
Prefrontal cortex follow-up	1.57 ± 0.41 (*N* = 25)	2.01 ± 0.25 (*N* = 10)	1.27 ± 0.12 (*N* = 15)	*t*(12) = 8.84***
Prefrontal cortex delta	0.09 ± 0.11***	0.15 ± 0.12**	0.05 ± 0.08*	*t*(15) = 2.36*
Parietal cortex baseline	1.46 ± 0.35 (*N* = 34)	1.81 ± 0.24 (*N* = 15)	1.19 ± 0.08 (*N* = 19)	*t*(17) = 9.75***
Parietal cortex follow-up	1.53 ± 0.38 (*N* = 25)	1.93 ± 0.22 (*N* = 10)	1.26 ± 0.16 (*N* = 15)	*t*(15) = 8.15***
Parietal cortex delta	0.10 ± 0.10***	0.16 ± 0.08***	0.06 ± 0.10*	*t*(22) = 2.56*
Lateral temporal cortex baseline	1.46 ± 0.33 (*N* = 34)	1.78 ± 0.24 (*N* = 15)	1.20 ± 0.06 (*N* = 19)	*t*(15) = 9.01***
Lateral temporal cortex follow-up	1.51 ± 0.37 (*N* = 25)	1.91 ± 0.24 (*N* = 10)	1.24 ± 0.10 (*N* = 15)	*t*(11) = 8.38***
Lateral temporal cortex delta	0.08 ± 0.09***	0.14 ± 0.09***	0.04 ± 0.06*	*t*(15) = 3.04**
Anterior cingulate baseline	1.74 ± 0.43 (*N* = 34)	2.16 ± 0.29 (*N* = 15)	1.40 ± 0.09 (*N* = 19)	*t*(16) = 9.68***
Anterior cingulate follow-up	1.69 ± 0.44 (*N* = 25)	2.18 ± 0.26 (*N* = 10)	1.37 ± 0.11 (*N* = 15)	*t*(11) = 9.37***
Anterior cingulate delta	0.03 ± 0.13	0.09 ± 0.15	–0.01 ± 0.10	*t*(14) = 1.93
Posterior cingulate baseline	1.88 ± 0.46 (*N* = 34)	2.30 ± 0.38 (*N* = 15)	1.55 ± 0.12 (*N* = 19)	*t*(16) = 7.43***
Posterior cingulate follow-up	1.82 ± 0.42 (*N* = 25)	2.22 ± 0.39 (*N* = 10)	1.56 ± 0.14 (*N* = 15)	*t*(11) = 5.11***
Posterior cingulate delta	0.02 ± 0.12	0.03 ± 0.15	0.02 ± 0.10	*t*(14) = 0.24
Precuneus baseline	1.67 ± 0.49 (*N* = 34)	2.17 ± 0.30 (*N* = 15)	1.28 ± 0.07 (*N* = 19)	*t*(15) = 11.27***
Precuneus follow-up	1.68 ± 0.49 (*N* = 25)	2.23 ± 0.25 (*N* = 10)	1.32 ± 0.11 (*N* = 15)	*t*(11) = 10.89***
Precuneus delta	0.08 ± 0.10***	0.14 ± 0.12**	0.04 ± 0.06*	*t*(13) = 2.29*

Stars denote significance levels in paired *t* test between baseline and follow-up measures: *p < 0.05, **p < 0.01, ***p < 0.00

Cognitive test performances of the two groups at baseline and follow-up are presented in Table 3. The mean Mini-Mental State Examination (MMSE) score was 27.2 ± 1.8 (range 24–30) and the mean logical memory I score (reflecting immediate recall) of Wechsler Memory scale revised (WMS-R, [20]) was 8.7 ± 3.6 and logical memory II score (reflecting delayed recall) was 6.6 ± 3.9 at baseline (see Table 3).

**Table 3:** Cognitive test results stratified by test time and amyloid status

	All	Amyloid positive	Amyloid negative	Group difference
MMSE baseline	27.2 ± 1.8 (*N* = 34)	26.8 ± 2.0 (*N* = 15)	27.5 ± 1.6 (*N* = 19)	*t*(26) = – 1.13
MMSE follow-up	26.0 ± 3.0 (*N* = 27)	24.4 ± 2.7 (*N* = 11)	27.2 ± 2.8 (*N* = 16)	*t*(22) = – 2.63*
MMSE delta	– 1.3 ± 2.8*	– 2.8 ± 2.4**	– 0.2 ± 2.6	*t*(23) = – 2.75*
Logical memory I baseline	8.7 ± 3.6 (*N* = 34)	7.3 ± 3.3 (*N* = 15)	9.8 ± 3.5 (*N* = 19)	*t*(31) = – 2.08*
Logical memory I follow-up	8.2 ± 4.0 (*N* = 26)	5.9 ± 3.2 (*N* = 10)	9.6 ± 3.9 (*N* = 16)	*t*(22) = – 2.60*
Logical memory I delta	– 0.8 ± 3.0	– 1.3 ± 2.3	– 0.5 ± 3.4	*t*(24) = – 0.72
Logical memory II baseline	6.6 ± 3.9 (*N* = 34)	5.5 ± 3.9 (*N* = 15)	7.5 ± 3.7 (*N* = 19)	*t*(30) = – 1.57
Logical memory II follow-up	6.1 ± 4.9 (*N* = 26)	3.5 ± 4.6 (*N* = 10)	7.8 ± 4.4 (*N* = 16)	*t*(18) = – 2.33*
Logical memory II delta	– 0.6 ± 3.8	– 1.6 ± 3.2	0.1 ± 4.1	*t*(22) = – 1.15
Fluency baseline	10.0 ± 5.3 (*N* = 34)	9.9 ± 6.8 (*N* = 15)	10.2 ± 3.8 (*N* = 19)	*t*(21) = – 0.15
Fluency follow-up	8.5 ± 3.5 (*N* = 26)	5.9 ± 2.3 (*N* = 10)	10.1 ± 3.1 (*N* = 16)	*t*(23) = – 3.87***
Fluency delta	– 1.1 ± 4.1	– 2.8 ± 5.0	0.0 ± 3.0	*t*(13) = – 1.60

For different tests delta refers to difference between baseline and follow-up in amyloid-positive and amyloid-negative groups separately and stars denote significance levels in paired t test between baseline and follow-up measures: *p < 0.05, **p < 0.01, ***p < 0.001

Patients with a positive ^18^F-Flutemetamol scan at baseline (*N* = 15; Table 3) showed a statistically significant decrement in MMSE, but not in the other cognitive measures over follow-up period, although numerically a decline in the scores of these tests was seen. Patients with negative baseline scans (*N* = 19) did not exhibit statistically significant mean decline in any of the cognitive measures. At baseline, amyloid-positive patients tended to perform more poorly than amyloid-negative patients in all cognitive tests, but statistically significant group-wise difference was observed only in the logical memory I test (Table 3). At follow-up, group-wise differences in cognition reached statistical significance (Table 3) for all cognitive tests. Moreover, the patients that were amyloid positive at baseline experienced more decline in MMSE, but not in other tests, as compared to the amyloid-negative patients.

**Fig. 2:**
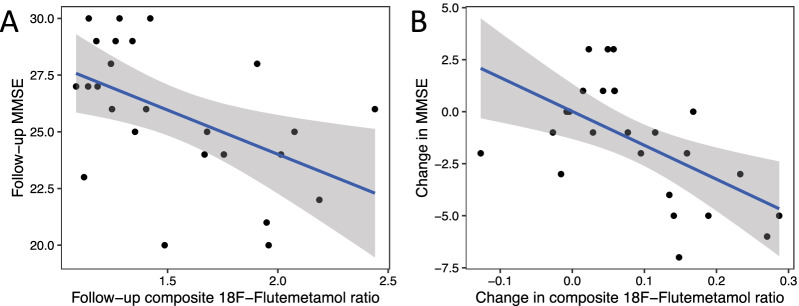
Composite 18F-Flutemetamol uptake ratio and MMSE score were negatively associated at follow-up (**A**), and in terms of change over time from baseline to the end of follow-up (**B**)

Associations between ^18^F-Flutemetamol uptake ratios and cognition were explored using Pearson’s correlation coefficients. Statistically significant associations were absent at baseline, but at follow-up, all cognitive measures were negatively associated with composite ^18^F-Flutemetamol uptake ratios (rs =  − 0.60, *p* < 0.01 to − 0.41, *p* = 0.047; Table 4, Fig. 2). Furthermore, increases in composite ^18^F-Flutemetamol uptake ratio were associated with decreases in MMSE and logical memory I test scores (rs =  − 0.57, *p* < 0.01 to − 0.46, *p* = 0.023; Table 4, Fig. 2). The negative change-change association in MMSE was manifested in amyloid-positive patients but not in the amyloid-negative patients (rs =  − 0.78, *p* < 0.01 and − 0.18, n.s., respectively).

**Table 4:** Associations between ^18^F-Flutemetamol composite scores and cognitive test results

	Pearson's correlation coefficient with [18F]Flutemetamol composite score
MMSE baseline	*r* = – 0.04 (*N* = 34)
MMSE follow-up	*r* = – 0.50* (*N* = 25)
MMSE delta	*r* = – 0.57**
Logical memory I baseline	*r* = – 0.31. (*N* = 34)
Logical memory I follow-up	*r* = – 0.41* (*N* = 24)
Logical memory I delta	*r* = – 0.46*
Logical memory II baseline	*r* = – 0.33. (*N* = 34)
Logical memory II follow-up	*r* = – 0.47* (*N* = 24)
Logical memory II delta	*r* = – 0.38
Fluency baseline	*r* = – 0.02 (*N* = 34)
Fluency follow-up	*r* = – 0.60** (*N* = 24)
Fluency delta	*r* = – 0.02

Pearson’s correlation coefficients and the corresponding significance levels are reported (.*p* < 0.1, *p < 0.05, **p < 0.01, ***p < 0.001)

## Discussion

In this study, we found that 15 of 34 (44%) aMCI patients had amyloid-positive ^18^F-Flutemetamol PET at baseline, and they showed statistically significant increase in ^18^F-Flutemetamol uptake during follow-up compared to patients with amyloid-negative baseline PET scan.

We also showed a statistically significant association between ^18^F-Flutemetamol increase and decrease in a general cognitive measure (MMSE) and a measure of episodic memory immediate recall (logical memory I) over time in individuals who were amyloid positive at baseline. There was also a trend-level association between increase in ^18^F-Flutemetamol uptake and decline in delayed episodic memory recall (logical memory II), but this did not reach statistical significance. In this study, the scores in the delayed recall measure (logical memory II) were already relatively low at baseline, leaving relatively little room for decline. Thus, a floor effect may explain why the association did not reach statistical significance. Contrary to our study, in earlier studies, the follow-up has been performed with clinical evaluation without amyloid PET scanning. In this study, as an extension to previous findings, following patients both clinically and with amyloid PET scans enabled us to analyze association between cognition and brain amyloid load over time.

Those patients who were amyloid positive at baseline performed more poorly in all cognitive tests both at the beginning of the study and at follow-up compared to amyloid-negative patients. Moreover, those patients with amyloid-positive PET scan at baseline had statistically significant decrease in MMSE test and also decline in other cognitive tests. Instead, patients with negative amyloid PET scan at baseline did not show statistically significant decrement in any cognitive tests. It is possible that the small increase (3–5.8%) in brain amyloid load during 3-year follow-up in the individuals who were amyloid negative at baseline could at least partially be due to the known age-related increase in brain amyloid load. Since the diagnosis of aMCI was based on clinical criteria without involvement of biomarkers, it is possible that the amyloid-negative aMCI patients at baseline probably have a different pathophysiology behind their MCI than those with amyloid-positive PET scan at baseline. In previous studies, the correlation between amyloid binding PET tracer retention and cognition has been generally fairly weak which is consistent with the notion that amyloid plaque distribution does not correlate well with clinical symptoms in AD. In addition, a few longitudinal ^11^C-PIB-PET publications indicate a lack of significant progression of ^11^C-PIB uptake in MCI and AD [19, 21–23]. AD patients apparently reach almost a plateau in ^11^C-PIB retention (and hence beta-amyloid accumulation) despite progression of their clinical symptoms. The lack of longitudinal increase in brain amyloid load in AD patients in previous studies suggests that amyloid tracer deposition most likely is an early event during the disease process. It has been suggested that amyloid biomarkers follow a sigmoid-shaped trajectory over time [24]. Therefore, in our study, aMCI patients with amyloid-positive PET scan were probably in the accelerating phase in the curve. At more advanced stage of MCI approaching AD stage, the increase in amyloid load would be less pronounced with probably weak association with the change in cognition. In the amyloid-negative aMCI patients, it is probable that the pathophysiology of their aMCI could be other than AD pathology, although theoretically they might turn amyloid positive later, but this is not supported by our findings with relatively short 3-year follow-up. In a previous study [25], 207 patients with memory problems were examined with ^18^F-Flutemetamol PET scan. 131 patients had diagnosis of MCI and 41 patients had diagnosis of AD before scanning. Most of the MCI and AD patients were ^18^F-Flutemetamol PET-positive (63 patients, 53% and 28 patients, 68%, respectively). In those patients with negative PET scan, the diagnosis changed to dementia due to non-AD disorders (including vascular dementia, progressive supranuclear palsy, dementia with Lewy bodies or frontotemporal dementia) or dementia of unclear etiology (dementia NOS) or led to retention/changed to MCI diagnosis.

^18^F-Flutemetamol and ^11^C-PIB are both brain amyloid binding PET ligands. They have been shown to differentiate between MCI patients and healthy controls [11, 26] and predict the progression of MCI to AD [5, 7, 13]. ^11^C-PIB is the first developed human amyloid imaging PET ligand and is widely used. ^18^F-Flutemetamol has some advantages over ^11^C-PIB. The half-life of ^18^F (ca. 110 min) enables wider accessibility for clinical and research use, enables shipment of the tracer from the production site to several, even remote, imaging sites, enables to scan several patients from a single synthesis batch and also enables longer PET acquisition times. The shorter ^11^C-PIB half-life (ca. 20 min) requires the use of on-site cyclotron and radiotracer production [16].

In this study, two different PET scanners were used. First, PET scanning of 14 patients was performed with ECAT EXACT HR + (CTI/Siemens, Knoxville, TN, US) camera. Later, both scans of 20 patients and a follow-up scan of two patients scans were performed with GE Discovery 690 (GE Healthcare, Waukesha, WI, US) PET scanner because at that time the other scanner was not in use anymore. Resolution and sensitivity of both scanners are very close to each other (see “PET and MRI imaging” section). In addition, the design of comparing the follow-up scan result to that of the baseline scan using the same scanner takes into account the possible difference in absolute uptake values between the scanners. Therefore, change of the scanner did not most probably have a significant effect on the results, especially since all but two patients were scanned with the same scanner both at baseline and follow-up.

## Conclusions

In this study, we found that in the early phase of aMCI, ^18^F-Flutemetamol uptake significantly increased during the follow-up in amyloid-positive patients and was associated with a decline in MMSE score. Some of our patients were amyloid negative also at end of the study. In those patients, the 18F-flutemetamol uptake did not increase significantly over time. Most probably their aMCI is due to pathology other that AD, but the 3-year follow-up is too short to determine this. Future studies with longer follow-up are needed.

## Data Availability

Due to the consent given by study participants and the high degree of identifiability, data cannot be made publicly available. Pseudonymized data may be shared with authorized researchers, upon researcher’s reasonable request, who have IRB/ethics approval and an institutionally approved study plan.
